# Potential of public health service screenings in kindergartens and schools for prevention reporting

**DOI:** 10.17886/RKI-GBE-2017-093

**Published:** 2017-08-30

**Authors:** Goetz Wahl

**Affiliations:** Saxony-Anhalt State Office for Consumer Protection, Department 2 – Hygiene, Unit 21 – Health and Hygiene Management

## Abstract

This article explores how screening examinations conducted by the public health service in Germany could contribute to identifying and reporting on health promotion and disease prevention needs in kindergartens and schools. In consideration of data protection guidelines and after obtaining prior consent from parents, screening examinations could be used to collect supplementary health relevant information in addition to routine data. The results could be relevant for an analysis of current needs, particularly regarding the local implementation of health promotion and disease prevention measures, as well as to ensure follow-up measures are backed by data.

## Background

In 2015, Germany adopted the Preventive Health Care Act, which aims to improve the co-ordination between current and future measures of health promotion and disease prevention as well as increase their efficiency. The federal framework recommendations issued within the framework of the Preventive Health Care Act identify three target areas and groups: Grow up healthy, Living and working healthy, Healthy ageing [1]. Moreover, they highlight three important factors that can contribute to the success of health promotion and disease prevention measures: identifying needs before measures are implemented, reaching target groups within their settings and committing to an evaluation based approach to prevention reporting.

Measures of health promotion and disease prevention are always implemented at the local level, i.e. in selected districts, town districts and/or specific institutions. Identifying needs and reporting on such measures must, therefore, primarily follow a local level approach. However, the necessary health data is often not available. Taking Saxony-Anhalt as an example, the following section illustrates how data collected by German public health service (Öffentlicher Gesundheitsdienst, ÖGD) during medical and dental screening examinations could help identify needs and contribute to reporting on health promotion and disease prevention projects for one of the previously mentioned target groups (children) in their setting (kindergartens and schools).

## The current situation in Saxony-Anhalt

In Saxony-Anhalt the ÖGD annually conducts health screenings for around 15,000 children of school-entry age [2], 11,000 third graders [3] and 11,000 sixth graders [4]. Around 150,000 children aged between 0 and 12 receive a dental check-up [5]. This amounts to 100%, 78%, 76% and 75% of all children in the respective age group registered as attending kindergarten or school. The methods used in these examinations and parent interviews are uniform across Saxony-Anhalt. As in most federal states, the results from examinations and interviews are encoded, entered using a computerised system and evaluated at the municipal and federal levels.

## Possible uses of screening data

A significant share of the variables recorded during routine data collection could potentially be used to identify needs and report on measures of health promotion and disease prevention that are directed at children. The use of data from dental screening to focus and evaluate measures of dental health promotion and disease prevention has been described elsewhere [5]. The following outlines an example of how data acquired through the medical screening of third graders could be used to monitor a (fictitious) project (Promotion of healthy physical activity and dietary habits in selected primary schools in the district of …), with initial measures being implemented when the children in question are still in first grade. The project is based on the assumption that the health relevant behaviour of primary school pupils (and their parents), who participate in a project of this nature over the course of several years, improves in the medium-term. Evidence for positive impacts, if any, would then be shown in data collected as pupils advance through primary school, for example, when they reach third grade.

In Saxony-Anhalt the data entry forms used in medical examinations include open fields where additional health relevant information concerning the children can be encoded and documented, and this could be used both to identify needs and supplement the evaluation of this type of measure. Such additional data collection would first have to be discussed with data protection officers. Furthermore, the physicians conducting the examinations would have to agree to the additional workload, and parents would have to give informed consent. In third grade, screening variables on body mass index, hypertension and musculoskeletal observations, together with data provided by the parents on the general health of their child as well as on sports club membership and the incidence of headaches, stomach aches and sleeping disorders could serve as physical activity and diet-related routine variables. Furthermore, third graders could be asked some questions on physical activity and dietary habits that could then be encoded and added to the open fields. Relevant questions could, for example, be: ‘How often do you exercise every week outside of school?’, ‘Where do you eat breakfast?’, and ‘How often do you eat a hot meal?’. Routine variables or additional questions could be selected to provide data on other issues relevant to disease prevention, such as addiction or media consumption. Collecting routine and additional information on specific issues for health promotion and disease prevention in this manner (Table 1) could serve to initially identify needs, for example, by highlighting particular districts and institutions where the need is greater. Furthermore, collected data would allow a follow-up to the project by measuring variables in selected intervention and control schools before and after intervention.

## Conclusion

The ÖGD screening examinations conducted in kindergartens and schools in Saxony-Anhalt generally provide an opportunity to identify needs (e.g. through pinpointing particularly vulnerable districts, town districts, institutions and target groups) and implement a data-backed follow-up in health promotion and disease prevention measures directed at children (e.g. dental health, physical activity, dietary habits, addiction and mental health).

## Figures and Tables

**Tabelle 1:** Example for a collection of variables relevant to prevention in annual screenings conducted by the public health service for third grade pupils in Saxony-Anhalt Own table

Concerns: physical activity, dietary habits, general health	
Variables	Finding/answer category
**Selected routine variables – screening**	
Body mass index (BMI)	Measurement: height/weight
Blood pressure	Measurement: systolic/diastolic blood pressure
Condition of the musculoskeletal system	Abnormality/medical referral/in treatment
**Anamnesis of selected routine variables (parent questionnaire)**	
Child’s general health	good/not satisfying
Frequent headaches	no/yes
Frequent stomach aches	no/yes
Frequent sleeping problems	no/yes
Membership of a sports club	no/yes
**Possible additional questions**	
Sports outside of school	< once/once or twice/≥ three times per week
Place where breakfast is eaten	At home/school/sometimes at home, sometimes at school
Hot meal frequency	daily/several times per week/only occasionally
